# Crosstalk Between Platelets and Microbial Pathogens

**DOI:** 10.3389/fimmu.2020.01962

**Published:** 2020-08-07

**Authors:** Conglei Li, June Li, Heyu Ni

**Affiliations:** ^1^Department of Immunology, University of Toronto, Toronto, ON, Canada; ^2^Toronto Platelet Immunobiology Group, University of Toronto, Toronto, ON, Canada; ^3^Department of Laboratory Medicine and Pathobiology, University of Toronto, Toronto, ON, Canada; ^4^Department of Laboratory Medicine, Keenan Research Centre for Biomedical Science, St. Michael's Hospital, Unity Health Toronto, Toronto, ON, Canada; ^5^Canadian Blood Services Centre for Innovation, Toronto, ON, Canada; ^6^Department of Physiology, University of Toronto, Toronto, ON, Canada; ^7^Department of Medicine, University of Toronto, Toronto, ON, Canada

**Keywords:** platelets, microbial pathogens, host immune responses, COVID-19, thrombosis

## Abstract

Platelets, small anucleate cells circulating in the blood, are critical mediators in haemostasis and thrombosis. Interestingly, recent studies demonstrated that platelets contain both pro-inflammatory and anti-inflammatory molecules, equipping platelets with immunoregulatory function in both innate and adaptive immunity. In the context of infectious diseases, platelets are involved in early detection of invading microorganisms and are actively recruited to sites of infection. Platelets exert their effects on microbial pathogens either by direct binding to eliminate or restrict dissemination, or by shaping the subsequent host immune response. Reciprocally, many invading microbial pathogens can directly or indirectly target host platelets, altering platelet count or/and function. In addition, microbial pathogens can impact the host auto- and alloimmune responses to platelet antigens in several immune-mediated diseases, such as immune thrombocytopenia, and fetal and neonatal alloimmune thrombocytopenia. In this review, we discuss the mechanisms that contribute to the bidirectional interactions between platelets and various microbial pathogens, and how these interactions hold relevant implications in the pathogenesis of many infectious diseases. The knowledge obtained from “well-studied” microbes may also help us understand the pathogenesis of emerging microbes, such as SARS-CoV-2 coronavirus.

## Introduction

Platelets are the second most abundant cells in human blood circulation (1, 2). Anucleate platelets are found only in mammals; in lower vertebrates, cells involved in hemostasis and blood coagulation are nucleated and termed thrombocytes (3, 4). Under physiological conditions, thrombopoietin (TPO) predominantly produced by the liver, via binding to the TPO receptor c-Mpl on megakaryocytes, is the major regulator of megakaryocyte differentiation and megakaryopoesis (5–7). Historically it is known that platelets are produced from their precursor megakaryocytes in the bone marrow of mammals (3, 8, 9). However, recent research surprisingly uncovered that platelets could also be generated by megakaryocytes in the lung of mice (10), although further validation is required in both murine and human studies. Additionally, the relative contribution of lung-generated platelets to total circulating platelets and whether they possess different function is still unclear (11). In extension to their traditional roles in haemostasis and thrombosis (12–15), recent studies suggest that platelets are also involved in many other physiological and pathophysiological processes, such as innate and adaptive immunity, angiogenesis, atherosclerosis, and tumor progression (2, 3, 16–22). We have previously compiled a comprehensive overview of the importance of platelets in modulating immune responses (23). In this review article, we mainly focus on the bidirectional interplay between platelets and microbial pathogens and the significant impact it has on the host responses.

**Figure 1:**
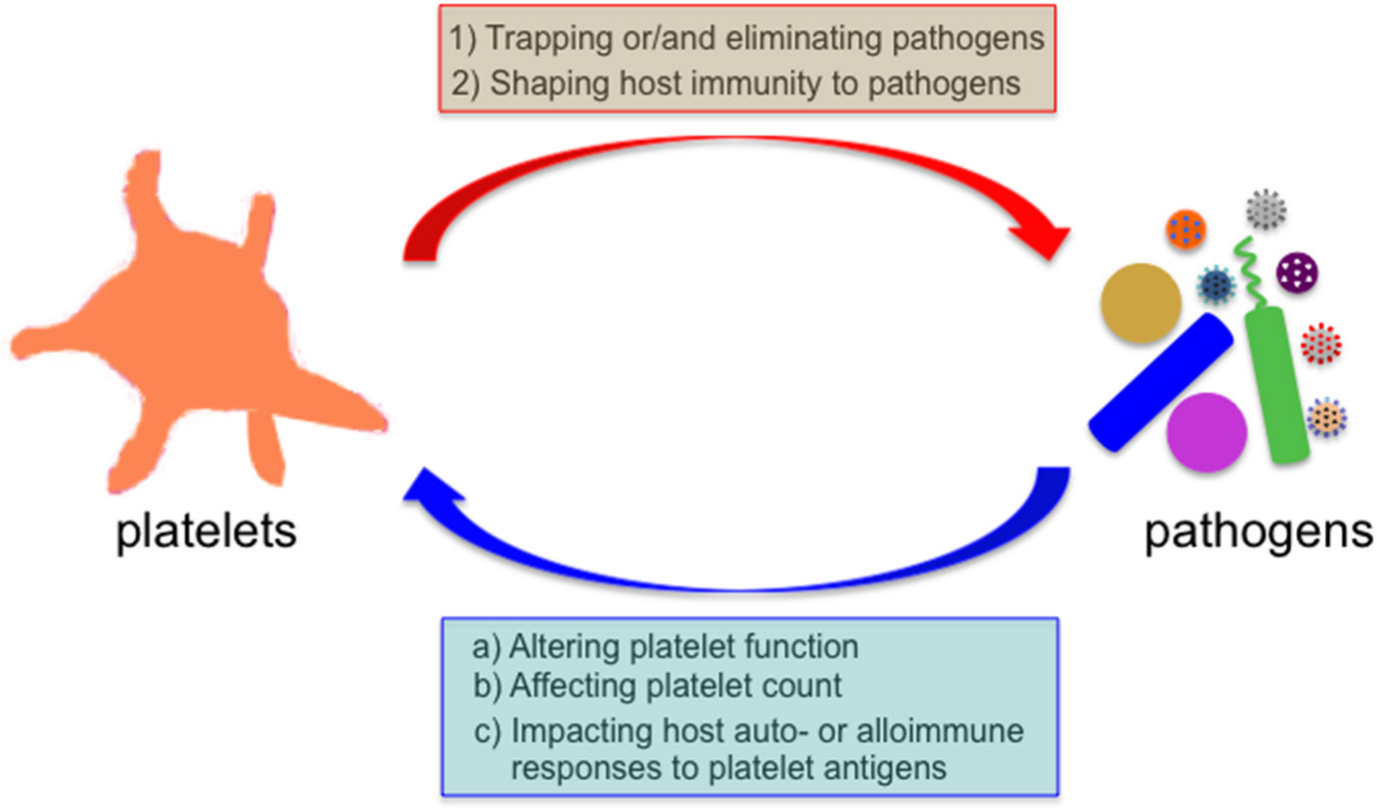
Bidirectional interaction between platelets and microbial pathogens. Microbes encounter platelets when they enter the mammalian blood circulation. Platelets exert their direct effects on microbial pathogens by either binding them and sequestering them thereby limiting their systemic dissemination or by directly eliminating them, and indirect effects by shaping the subsequent host immune response to these invaders. Reciprocally, many invading microbes can alter platelet count or/and function, and impact the host auto- and alloimmune response to platelet antigens in several immune-mediated diseases.

Infectious diseases are unresolved challenges to human health, and remain as one of leading causes of morbidity and mortality worldwide, especially in resources-limited countries (https://www.who.int/news-room/fact-sheets/detail/the-top-10-causes-of-death). Microorganisms encounter platelets when they enter the mammalian blood circulation. Platelets can directly bind to many pathogens (e.g., bacteria, viruses, and parasites), or pathogen-IgG immune complexes via Fc receptors expressed on platelets (24–26). This platelet-pathogen interaction has functional consequences on both platelets and pathogens (Figure 1). Reduced levels of circulating platelets are commonly observed in patients with infectious diseases, and the underlying mechanisms vary depending on specific pathogens (18, 19, 25, 26). In addition, it has been demonstrated that reduced platelet counts in patients or mice are associated with increased susceptibility of the host to infections (27–30). Sepsis is a life-threatening inflammatory syndrome caused by a dysregulated host response to infection (31), and it has been demonstrated that sepsis altered the transcriptional and translational profiles of platelets in both humans and mice (32). Although the evolutionary pressure to drive the pathogens to develop various strategies to target platelets is not well-understood, one possibility is that platelets may protect the host from certain invading pathogens.

## Versatile Roles of Platelets in Physiology and Pathobiology

### Role of Platelets in Haemostasis and Thrombosis

Platelet adhesion, activation and aggregation at the damaged vessel endothelium are critical for bleeding arrest (12–15). Platelet surface glycoprotein receptor, GPIbα, via interacting with von Willebrand factor (VWF; anchored on collagen in the injured vessel wall), initiates platelet adhesion, particularly under the high shear conditions (14, 15, 33, 34). The GPIbα-VWF interaction is also critical for endovascular growth of occlusive thrombi at sites of arterial stenosis where blood flows with wall shear rates that may exceed 40,000 s^−1^, corresponding to shear stresses exceeding 1,600 Pa (35). The glycoprotein GPIIbIIIa (αIIbβ3 integrin), can also contribute to platelet adhesion under the lower shear conditions. This abundant platelet integrin is essential for both fibrinogen-dependent and fibrinogen-independent platelet aggregation (34, 36–39). Interestingly, in addition to the platelet accumulation (platelet adhesion and aggregation, the first wave of haemostasis), we recently found that the plasma fibronectin can rapidly deposit onto the injured vessel wall and mediate a “protein wave of hemostasis,” which occurs even earlier than the first wave of haemostasis (40, 41). Platelets may release their plasma fibronectin content from α granules and contribute to this protein wave of hemostasis, which is likely a compensatory mechanism for heamostasis in fibrinogen-deficient mice and humans since their platelet fibronectin levels increase 3–5-fold (34, 42, 43). Notably, activated platelets can promote the cell-based generation of thrombin that markedly enhances the blood coagulation (the second wave of haemostasis) leading to the generation of polymerized fibrin (2, 14, 44). Thus, platelets contribute to all three waves of haemostasis, which may directly or indirectly affect the dissemination of micropathogens *in vivo*.

It has been well-understood that deficiencies in platelet adhesion/aggregation or the coagulation system are linked with various bleeding disorders (2, 36, 45). However, inappropriate formation of platelet plug may lead to thrombosis, and thrombosis in the cerebral or coronary arteries is the major cause of morbidity and mortality worldwide (46–48). Moreover, it has been recognized that thrombus formation in the placenta can lead to fetal loss during pregnancy in several disease conditions (49), such as antiphospholipid syndrome (50, 51).

### Role of Platelets in Innate and Adaptive Immunity

As platelets contain both pro-inflammatory and anti-inflammatory molecules, platelets can interact with many immune cells (e.g., dendritic cells, neutrophils, and lymphocytes), which can shape both innate and adaptive immunity (3, 16, 17, 21, 23). In addition, platelets are involved in the development of lymphatic vessels, the critical network facilitating immune cell trafficking and surveillance (52, 53). Platelets achieve this via the binding of platelet C-type lectin-like receptor 2 to podoplanin on lymphatic endothelial cells, leading to the separation of lymphatic vessels from blood vessels during embryonic development (54–56).

Platelets contribute to the host innate immunity in various ways. Platelets express the functional pathogen recognition receptors, such as Toll-like receptors (TLRs) (TLRs 1-10 in human platelets and TLRs 1-8 in murine platelets), and Nod-like receptor 2 (19, 25, 57, 58). Platelets contain many pro-inflammatory molecules (e.g., CD40 and serotonin), cytokines (e.g., IL-1) and chemokines (e.g., CCL3, CXCL4, and CCL5), and antimicrobial factors (e.g., kinocidins and defensins) in their granules (3, 19). In addition, platelets express several functional chemokine receptors, such as CCR3, CCR4, and CXCR4 (59). Platelets can also shed microparticles that are capable of transporting inflammatory molecules (e.g., CD40L and IL-1) to inflammatory cells (16, 60). Interestingly, platelets also contain multiple anti-inflammatory molecules and cytokines, such as transforming growth factor-β (TGF-β) (3). It has been shown that platelet-derived TGF-β diminishes the anti-tumor activity of natural killer (NK) cells (20, 61).

Platelets also modulate adaptive immune response of the host. Activated platelets express CD40L on their surface, which plays a key role in supporting antibody isotype switching (e.g., from IgM to IgG) and enhancing CD8^+^ T cell function (62, 63). Upon platelet activation, P-selectin is translocated from the α-granule to the platelet surface (64). P-selectin, via interacting with peripheral node addressin on high endothelial venules and P-selectin glycoprotein ligand-1 on lymphocytes, mediates the rolling and recruitment of lymphocytes to peripheral lymph nodes (65). And platelet-derived TGF-β was shown to inhibit the cytotoxic T cell response in the tumor microenvironment (66), and might improve function of regulatory T cells (67).

TGF-β is a key factor in IgA isotype switching (68). Since IgA plays an important role in controlling the homeostasis of gut microbiota (68), and preventing pathogen invasion at mucosal sites (69), it remains to be investigated whether platelet TGF-β contributes to the production of intestinal IgA. In addition, TGF-β is critical for the differentiation of regulatory T cells under non-inflammatory conditions, in both mice and humans (70–72).

## Effects of Platelets on Microbial Pathogens

**Figure 2:**
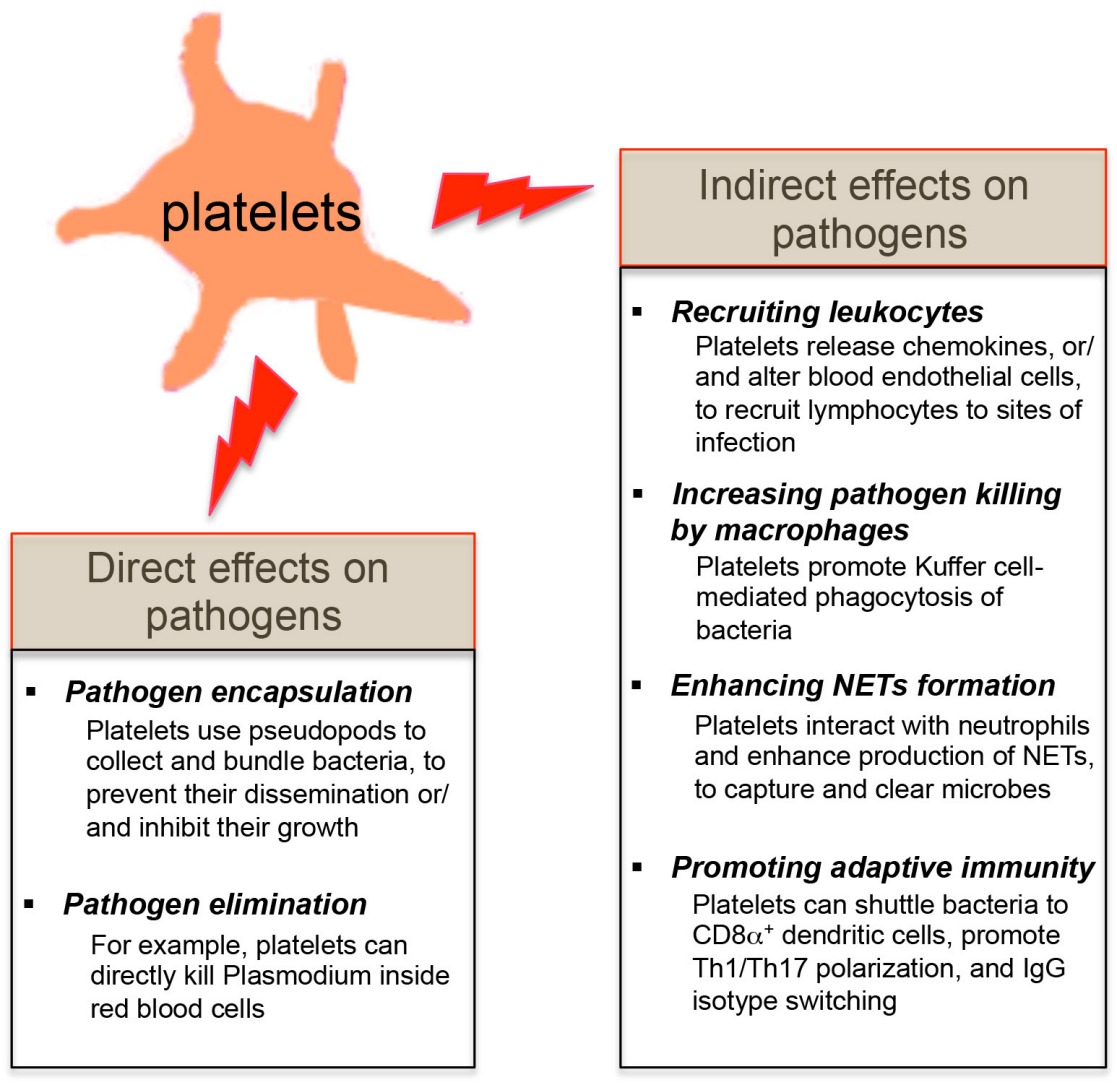
Effects of platelets on microbial pathogens. The direct effects of platelets on microbial pathogens include pathogen encapsulation and elimination. Platelets also exert the indirect effects on microbial pathogens by shaping the innate and adaptive immune responses of the host against these invaders.

Since platelets contain many pro-inflammatory molecules, and reduced platelet counts in patients or mice are linked with the host's susceptibility to infections (3, 23, 27, 28, 30), it suggests that platelets may protect the host from certain microbial infections. Platelets are involved in the early detection of invading microorganisms and are actively recruited to sites of infection (18, 19, 25). Review of recent literatures shows that platelets exert their direct effects on microbial pathogens by either binding them and sequestering them thereby limiting their systemic dissemination or by directly eliminating them (Figure 2). Platelets also have indirect effects on microbial pathogens by shaping the subsequent innate and adaptive immunity of the host to these invaders (Figure 2).

### Direct Effects of Platelets on Pathogens

In the context of *Staphylococcus aureus* (*S. aureus*) infection, platelets bind *S. aureus* and use the pseudopods to encapsulate the bacteria (73). This ability of platelets to collect and bundle bacteria [e.g., *S. aureus, Escherichia coli* (*E. coli*) and *Listeria monocytogenes* (*L. monocytogenes*)] may trap these bacteria, limit their dissemination within the bloodstream and present them to phagocytes (74, 75). Moreover, α-toxin derived from *S. aureus* stimulated human platelets to release β -defensins, which significantly retarded the growth of two strains of *S. aureus* isolated from patients with sepsis (73).

In addition to pathogen trapping, platelets can kill certain pathogens. *Plasmodium. falciparum* is the most common species that cause malaria in humans. In the infected host, *Plasmodium* invades red blood cells in the bloodstream and replicate until erythrocytes burst. It has been demonstrated platelets can bind *Plasmodium*-infected erythrocytes and directly kill *Plasmodium* inside red blood cells both *in vitro* and *in vivo* (28, 76). Subsequent studies revealed that the chemokine platelet factor 4 (also known as CXCL4) released from platelets plays a key role in this platelet-mediated parasite destruction (77, 78). In addition, platelets can secrete many antimicrobial factors including defensins to inhibit the growth of bacteria and viruses (19). Notably, human platelets and megakaryocytes express the antiviral immune effector molecule: interferon-induced transmembrane 3 (IFITM3) (79). It has been recently demonstrated that viral infections (e.g., influenza and dengue viruses) upregulated the expression of IFITM3 on platelets and megakaryoctyes, eliciting rapid antiviral immunity, and that megakaryocytes were capable of limiting viral infections in both megakaryocytes and hematopoietic stem cells via secretion of type I interferons (79).

However, it is important to note that some viruses [e.g., Dengue virus, human immunodeficiency virus (HIV), and hepatitis C virus (HCV)], which can be actively engulfed by platelets and induce platelet activation through TLR signaling, may also utilize platelets to disseminate through the entire body of host (80–83). Therefore, the protective role of platelets against viruses may be context-dependent.

### Indirect Effects of Platelets on Pathogens

In addition to the direct effects on pathogens, platelets can shape the host immune responses to invading pathogens and the involved mechanisms are summarized as follows (Figure 2):

#### Recruiting Leukocyte to Sites of Vascular Invasion

Platelets can utilize the functional pattern recognition receptors expressed on their surface to sense the intravascular pathogens, and release various chemokines (e.g., CCL3, CXCL4, and CCL5) to recruit leukocytes to sites of vascular invasion (18). In addition, activated platelets use CD40L to trigger the inflammatory reaction on CD40-expressing vascular endothelial cells, leading to increased expression of the adhesion molecules (e.g., vascular cell adhesion molecule 1 and intercellular adhesion molecule 1) and secretion of proinflammatory cytokines (e.g., CCL2) by endothelial cells (84). This phenotypic alteration of vascular endothelial cells may further promote the recruitment of leukocytes at sites of infection (85, 86).

Activated platelets can also directly interact with leukocytes, forming platelet-leukocyte conjugates, and this interaction is largely mediated by P-selectin on activated platelets and P-selectin glycoprotein ligand 1 on leukocytes (47). The platelet-leukocyte triggers the activation of leukocytes and their increased expression of β1 and β2 integrin, leading to enhanced adhesion of leukocytes to vascular endothelial cells (87). In addition, activated platelets already deposited at sites of infection can act as docking platforms for leukocyte recruitment (47). More importantly, activated platelets deposit chemokines CXCL4 and CCL5 on the surface of vascular endothelial cells, instructing the extravasation of leukocytes at sites of infection (88, 89).

#### Increasing Pathogen Elimination by Macrophages

In the liver, the tissue-resident macrophages, Kupffer cells, play a key role in the innate defense against blood-borne pathogens. Wong et al. showed that Kupffer cells act as docking platforms for both bacteria and platelets. Platelets formed aggregates around the bacteria that are bound to Kupffer cells, and promoted Kupffer cell-mediated phagocytosis of these bacteria (90).

#### Enhancing Formation of Neutrophil Extracellular Traps (NETs)

During Gram-negative bacterial infections, platelets actively contribute to NETs formation (29, 91). Platelet TLR4 is capable of detecting intravascular TLR4 ligands [e.g., lipopolysaccharide (LPS)], inducing platelet binding to neutrophils. This TLR4-dependent platelet-neutrophil interaction results in robust neutrophil activation and production of NETs, which are DNA-based structures capable of capturing and eliminating microbes from the bloodstream (29, 77). Platelet depletion *in vivo* significantly impairs NETs formation and bacterial clearance (29, 92).

#### Promoting Adaptive Immune Response to Pathogens

Antigen acquisition by dendritic cells is critical for generation of the cytotoxic CD8^+^ T cell response against intracellular pathogens (93). Verschoor et al. found that platelets could actively bind *L. monocytogenes* in the circulation and shuttle this subset of gram-positive bacteria to splenic CD8α^+^ dendritic cells, enhancing anti-bacterial CD8^+^ T cell expansion (94). In addition to affecting antigen presentation, platelets have been shown to promote the polarization of Th1 and Th17 cells, and modulate the balance of regulatory and non-regulatory T cells (95, 96). Furthermore, platelet-derived CD40L alone is sufficient to induce IgG isotype switching against adenovirus (62), but it remains to be investigated whether platelet CD40L also promotes antibody class switching to other immunologobulin isotypes (e.g., IgA), since antibody class switching to different isotypes involves distinct DNA repair pathways (97).

Conversely, platelet antimicrobial responses may be detrimental to the host if they are dysregulated. For example, it has been reported that NETs formation contributed by platelets that were activated by microbial derived products could cause the injury to blood endothelial cells due to the many proteases contained within NETs (29), which can directly act as a scaffold and stimulus for thrombus formation (98).

## Effects of Microbial Pathogens on Platelets

**Figure 3:**
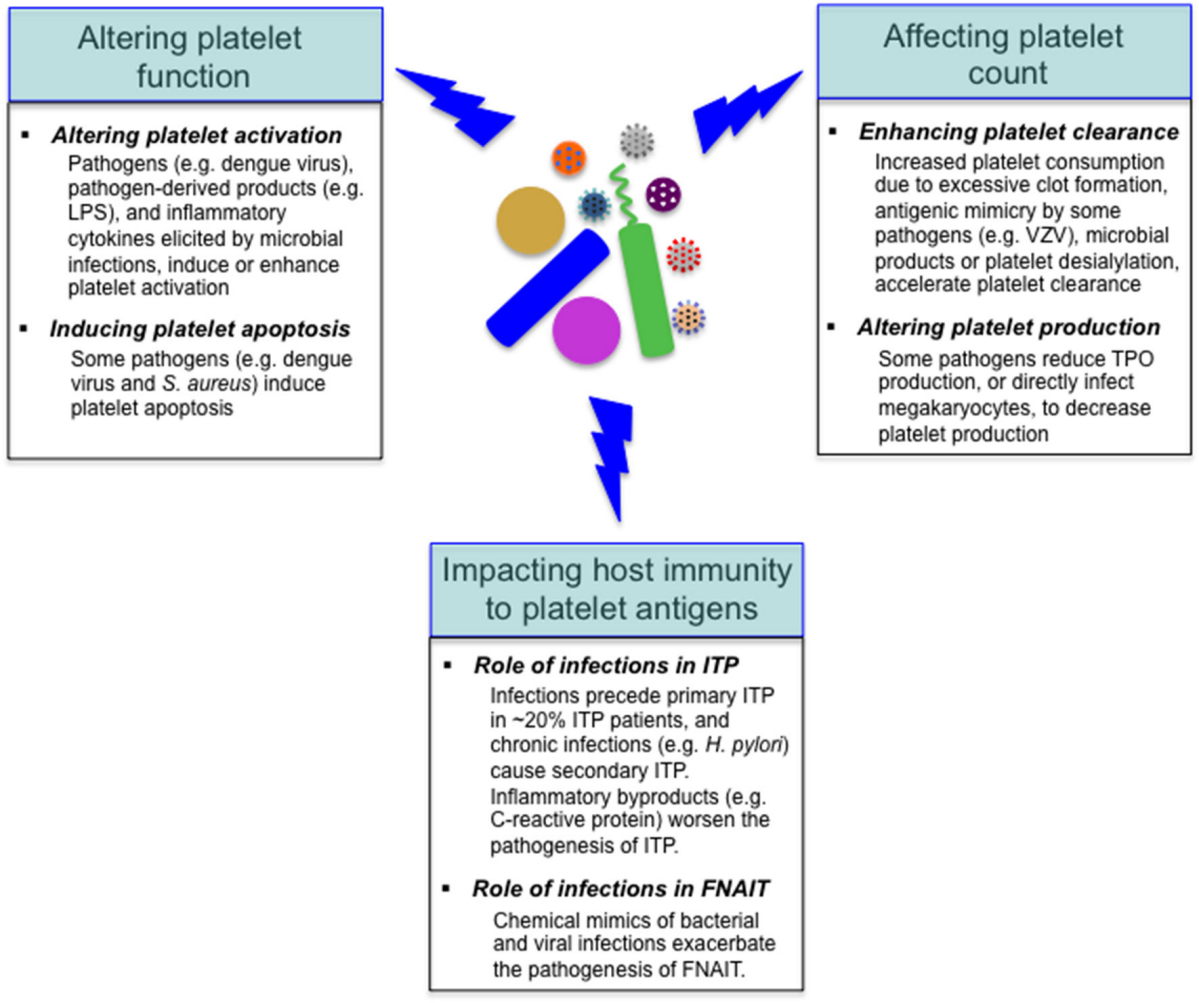
Effects of microbial pathogens on platelets. Many invading microbes can alter platelet function, leading to platelet activation or/and apoptosis. Reduced platelet count is a common feature with some infectious diseases, and the underlying mechanisms include accelerated platelet clearance and impaired platelet production. In addition, microbial pathogens impact the host autoimmune (e.g., in ITP) and alloimmune (e.g., in FNAIT) response to platelet antigens. VZV, varicella zoster virus.

As mentioned above, reduced platelet count is a common feature with some infectious diseases, and the underlying mechanisms vary depending on specific pathogens (18, 25, 26). Considering the important role of platelets in the regulation of host immunity, it is not surprising that various pathogens target platelets in the course of infections. Many invading pathogens can directly or indirectly target platelets in the host, altering platelet function or/and count (Figure 3); in addition to these alterations, it has been shown that viral infections (e.g., dengue and influenza viruses) and sepsis can markedly alter the platelet transcriptome (32, 79). Furthermore, microbial pathogens impact the host autoimmune and alloimmune response to platelet antigens in several immune-mediated diseases, such as immune thrombocytopenia, and fetal and neonatal alloimmune thrombocytopenia (99–101) (Figure 3).

### Impact of Microbial Pathogens on Platelet Function

The interaction between microbial pathogens and platelets can lead to alteration of platelet function (i.e., platelet activation and apoptosis) (Figure 3). Activated platelets can trigger the coagulation system, leading to excessive clotting (102, 103), which may exacerbate the symptoms caused by microbial infections and thus may be detrimental to the host.

#### Altering Platelet Activation

The capacity to trigger platelet activation is a well-known feature for many pathogens. For example, LPS purified from Gram-negative bacterium *E. coli*, via interacting with TLR4, induces platelet activation both *in vitro* and *in vivo* (58, 104), and direct interaction between *E. coli* and platelets has also been observed *in vivo* (75). Dengue virus, which causes hemorrhagic fever in around 10% infected patients, directly bind platelets via multiple receptors (e.g., DC-SIGN, heparin sulfate proteoglycan receptors and TLR-4), and activate platelets, triggering the conformational activation of platelet αIIbβ3 integrin, the translocation of P-selectin to platelet surface and the release of pro-inflammatory molecules (e.g., IL-1β) (25, 105, 106). In addition, microbial infections cause the release of inflammatory cytokines in the host (31, 107), and these cytokines (e.g., TNF-α) were shown to enhance platelet activation *in vivo* (108). And for some pathogens (e.g., influenza virus), anti-microbial antibodies form the immune complexes with pathogens and activate platelets via Fc receptors (18, 109).

It has been shown that the secreted products by *S. aureus*, such as α-toxin and Staphylococcal superantigen-like 5, can directly activate platelets (110, 111). Interestingly, the lipoteichoic acid secreted by *S. aureus* can inhibit platelet activation and aggregation (112). Thus, the effects of microbial pathogens on platelet function is dependent on microbial strain or/and the microbial products.

#### Inducing Platelet Apoptosis

Once activated, platelets undergo apoptosis (25). In addition, some pathogens (e.g., pathogenic *E. coli* and *S. aureus*) are found to directly induce platelet apoptosis through degradation of anti-apoptotic Bcl-xL protein (113). Platelet apoptosis induced by microbial pathogens (e.g., dengue virus) not only reduces mitochondrial potential, but also increases the surface exposure of phosphatidylserine that potentially triggers the activation of coagulation system (106, 114).

### Impact of Microbial Pathogens on Platelet Count

Reduced platelets in the context of infectious diseases can be due to enhanced platelet clearance or/and altered platelet production (Figure 3).

#### Enhancing Platelet Clearance

As mentioned above, some microbial pathogens can activate the platelet and coagulation system, leading to thrombosis (18). Exaggerated thrombus formation, especially within the setting of sepsis-associated disseminated intravascular coagulation, may excessively consume platelets, resulting in reduced circulating levels (31, 115, 116). Secondly, platelet clearance may be enhanced through collateral stimulation of the immune system by some microbial pathogens [e.g., varicella zoster virus, HIV, HCV, and *Helicobacter pylori* (*H. pylori*)] (117–121). For example, thrombocytopenia in children following varicella zoster virus infection first described antigenic mimicry for some microbial pathogens that encompass host generation of cross-reactive antibodies to certain glycoproteins (e.g., GPIIIa) on the platelet surface, resulting in accelerated platelet clearance (117). Third, direct platelet-bound microbial products (e.g., LPS) or inflammatory byproducts (e.g., C-reactive protein) could enhance antibody mediated phagocytic responses (122–124).

Microbial induced platelet clearance can also occur via removal of terminal sialic residues of the abundantly expressed platelet surface glycans. Scavenging of host sialic residues by microbial pathogens increases immune evasion and assists in survival and dissemination (125, 126). Direct cleavage of platelet sialic residues by pathogen-derived neuraminidase has been reported in bacterial, and parasitic infections (127). Indirectly, pathogens could induce platelet desialylation mediated by platelet-derived neuraminidase, as was reported with dengue virus infection (128). By either mechanism, loss of terminal sialic residues not only leads to rapid platelet clearance via lectin receptors predominantly in the liver (129), but also potentiates platelets to hyperactivity contributing to pathological disseminated intravascular coagulopathy and thrombotic complications of sepsis (130–132). Although, animal models and preliminary human studies demonstrate sialidase inhibitors or hepatic lectin receptor Ashwell-Morell inhibitors can ameliorate coagulopathies and thrombocytopenia in microbial infections (133, 134), other lectin receptors such as the recently identified Kupffer macrophage galactose lectin receptor may also contribute (135). Likely there are multiple and redundant receptor/ligand interactions that mediate clearance of desialylated or desialylation activated platelets.

#### Altering Platelet Production

Depending on the specific pathogens, there are several means by which invading microbes can negatively impact the platelet production by megakaryocytes in the bone marrow. For example, HCV can interfere with TPO production by damaging the liver tissue (136). Some pathogens (e.g., dengue virus and HIV) can directly infect megakaryocytes or their precursors, or alter the bone marrow microenvironment, leading to the defective platelet production in bone marrow (137–140).

However, it is important to note that inflammatory cytokines (e.g., TNF-α and IL-6) induced by certain microbial infections are capable of enhancing platelet production by triggering acute emergency megakaryopoiesis (18, 141). Thus, the impact of microbial infections on platelet production is context-dependent.

In addition to the effects on platelet count and function, microbial pathogens impact the host auto- and alloimmune response to platelet antigens in several immune-mediated diseases, such as immune thrombocytopenia (ITP), and fetal and neonatal alloimmune thrombocytopenia (FNAIT) (99–101) (Figure 3).

#### Role of Infections in ITP

ITP is an autoimmune disorder in which an abnormal immune response develops against one's own platelets, leading to autoantibody-induced platelet/megakaryocyte destruction and suppressed platelet production, and an increased risk of bleeding (3, 99, 142–145). In adult ITP patients, detectable antibody reactivity against GPIIbIIIa and GPIb/IX predominate (60–70%) (99, 142, 146). However, it is uncommon for patients to possess single antibody specificities, other glycoprotein targets including GPV, GPIV, and GPIa/IIa are often detected (147–149). Moreover, extensiveness of anti-glycoprotein antibody repertoire has been correlated with more severe disease (147). The anti-platelet antibodies not only accelerate platelet clearance mediated by splenic macrophages and hepatic Kupffer cells (135, 150, 151), but also inhibit the development of bone marrow megakaryocytes and promote their apoptosis, thus inhibiting platelet production (3, 99, 129, 142, 152). In addition to anti-platelet autoantibodies, cytotoxic CD8^+^ T cells, and regulatory CD8^+^ T cells might also contribute to the pathogenesis of ITP (99, 142, 153–158). Cytotoxic CD8^+^ T cells were shown to directly lyse platelets, induce the apoptosis of platelets, and inhibit platelet production by megakaryocytes (153, 159, 160). It has been reported that the frequency or/and function of regulatory CD4^+^ T cells were defective in the circulation of ITP patients (161–169), and interestingly, the TGF-β level was also reduced in these patients (161, 170, 171). It has been reported that peripheral deficiency of regulatory CD4^+^ T cells might be caused by their retention in the thymus in murine model of ITP (172), although it remains to be investigated whether this mechanism is also present in ITP patients. The therapies (e.g., steroids and B cell depletion) that improve platelet counts also restored the frequency or/and function of regulatory CD4^+^ T cells in the periphery (67, 163, 166, 169, 173), and the level of circulating TGF-β in ITP patients (170, 171), although it remains to be investigated whether the improvement of regulatory T cells is due to changes in circulating TGF-β (3).

Chronic infections (e.g., HIV, HCV, and *H. pylori*) can cause secondary ITP, in which antimicrobial antibodies cross-react with platelets, leading to platelet destruction (174). Acute infections have long been suspected as triggers that initiate the pathogenesis of primary ITP, but in most acute ITP cases, the specific pathogen could not be identified (175). Retrospective studies suggested that infectious events (e.g., viral and fungal infections) precede the development of primary ITP in around 20% ITP patients (176), but future definitive studies are required to confirm the causal relationship between the specific pathogen(s) and initiation of primary ITP, and to identify the underlying mechanisms (151, 152). Furthermore, it has been demonstrated that infections during ITP worsened the pathogenesis of primary ITP and the therapeutic response to platelet transfusions, but the underlying reasons were unclear (30).

Since inflammation induced hemorrhage in thrombocytopenic mice (177), it is possible that inflammation associated with microbial infections may aggravate the bleeding risk in thrombocytopenic patients (e.g., ITP). C-reactive protein is markedly upregulated during acute infections and inflammation (178), and it has been shown that C-reactive protein, via binding to platelet phosphorylcholine residues, enhanced the IgG-mediated phagocytic responses against platelets and thereby thrombocytopenia, which has implications in the pathogenesis of both ITP and FNAIT (123, 124). In addition to ITP, infections also play an important role in the pathogenesis of heparin-induced thrombocytopenia, in which pathogenic antibodies to the complexes of platelet factor 4 (PF4) and heparin develop post-heparin exposure, leading to life-threatening complications of thrombocytopenia and thrombosis (179, 180). It has been demonstrated that PF4 bound to various bacteria, induces the generation of antibodies that could cross-react with the major antigen in PF4/heparin complex, resulting in heparin-induced thrombocytopenia (181, 182).

#### Role of Infections in Alloimmune Thrombocytopenia

FNAIT results from the development of maternal alloantibodies targeting paternally derived antigens on fetal platelets during pregnancy, and these maternal antibodies cross the placenta and destroy fetal or neonatal platelets, leading to bleeding disorders (183–189). Similar to ITP, most of the reported FNAIT cases are characterized by maternal alloantibodies to platelet GPIIbIIIa (183–185, 190). In contrast, there are very few reported cases of FNAIT with anti-GPIbα complex antibodies (191–195), which is different from the 20 to 40% prevalence of anti-GPIbα antibodies in ITP patients (99, 142). To gain new insights into the pathogenesis of FNAIT, our laboratory established animal models of FNAIT using β3^−/−^ and GPIbα^−/−^ mice, respectively (187, 188, 196, 197). We observed neonatal thrombocytopenia and severe bleeding disorders (e.g., intracranial hemorrhage) in the heterozygous pups from wild-type (WT) platelet immunized β3^−/−^ dams, which recapitulated FNAIT in humans (187, 196). In contrast, miscarriage unexpectedly occurred in most of the anti-GPIbα-mediated FNAIT, which is far more frequent than that mediated by anti-β3 antibodies (49). Besides miscarriage, maternal immune response against fetal platelet antigens caused intrauterine growth restriction to fetuses due to placental abnormalities in animal models of FNAIT (197).

The roles of bacterial/viral infections in the pathogenesis of FNAIT were unclear. To test whether bacterial infection contributed to FNAIT, we utilized LPS to mimic Gram-negative bacterial infection, and co-administered it with low-dose WT platelet antigens to GPIbα^−/−^ and β3^−/−^ mice (100). We found that LPS co-administration significantly boosted the production of anti-GPIbα and anti-β3 antibodies, and miscarriage occurred in most of these co-stimulated GPIbα^−/−^ and β3^−/−^ mice, while miscarriage infrequently occurred in the dams immunized with low-dose WT platelets alone. Furthermore, we utilized Poly I:C to mimic viral infections, and observed that co-injection of Poly I:C and WT platelets also enhanced production of anti-GPIbα antibodies in GPIbα^−/−^ mice and the severity of FNAIT (100). However, it remains to be investigated whether live bacterial or viral infections indeed exacerbate the pathogenesis of FNAIT. Overall, our data suggested that both bacterial and viral infections were likely to be involved in the pathogenesis of FNAIT in animal models, but it warrants further studies to test whether this is also the case for human FNAIT patients.

The effects of microbial infections in the pathogenesis of FNAIT may be also translatable to another alloimmune thrombocytopenia: post-transfusion purpura, in which anti-platelet alloantibodies develop against transfused platelets from genetically distinct donors (3).

## Future Perspectives

Our understanding of platelet functions beyond haemostasis and thrombosis has dramatically expanded in the past years. Accumulating evidence indicates that platelets play an important role in the host immunity against microbial infections, and future discoveries will undoubtedly uncover more versatile features of platelets. The interaction between platelets and microbial pathogens are bidirectional, as this interaction causes the biological consequences on both platelets and microbes (Figure 1). The knowledge we obtained from these “well-studied” microbes may also help us understand the pathogenesis of emerging microbes, such as severe acute respiratory syndrome coronavirus (SARS-CoV-2). The SARS-CoV-2 infection causes the pandemic coronavirus disease 2019 (COVID-19) in humans, but the pathogenesis of COVID-19 is still largely unclear (198, 199). Thrombocytopenia has been observed in around 5–36% of COVID-19 patients (198, 200), and two recent meta-analysis studies with COVID-19 patients revealed that severe reduction in platelet counts might be a poor prognostic marker for this life-threatening disease (201, 202). Importantly severely ill COVID-19 patients exhibit profound hypercoagulable states (203, 204), and excessive clotting has been observed in severely ill COVID-19 patients (205–207). Is the thrombocytopenia in severe COVID-19 cases caused by the platelet hyperactivities and consumption during micro-thrombi formation? Do the hypercoagulable states synergize with platelet activation, which provide phosphatidylserine, propel the cell-based thrombin generation (44), and lead to thrombosis? Do platelets release/synthesize their cytokines and contribute to the cytokine storm in COVID-19 patients? Do platelets contribute to the immune response against SARS-CoV-2? Finally, are platelets friends or foes or able to switch their roles during SARS-CoV-2 infection? All these questions are important and warrant further investigations.

Overall, we believe that understanding the interactions between platelets and microbial pathogens will shed light on the pathogenesis of infectious diseases and that modulation of platelet-pathogen interactions could provide new therapeutic avenues.

## Author Contributions

CL designed and wrote most of the paper. JL wrote and edited the manuscript. HN was the principal investigator who designed and wrote the paper. All authors contributed to the article and approved the submitted version.

## Conflict of Interest

The authors declare that the research was conducted in the absence of any commercial or financial relationships that could be construed as a potential conflict of interest. The reviewer RK declared a past co-authorship with one of the authors HN to the handling editor.

## References

[1] Michelson M Platelets. Academic Press: London (2019).

[2] Xu XR Zhang D Oswald BE Carrim N Wang X Hou Y. Platelets are versatile cells: new discoveries in hemostasis, thrombosis, immune responses, tumor metastasis and beyond. Crit Rev Clin Lab Sci. (2016) 53:409–30. 10.1080/10408363.2016.1200008 27282765

[3] Semple JW Italiano JE Jr Freedman J. Platelets and the immune continuum. Nat Rev Immunol. (2011) 11:264–74. 10.1038/nri2956 21436837

[4] Wang Y Andrews M Yang Y Lang S Jin JW Cameron-Vendrig A. Platelets in thrombosis and hemostasis: old topic with new mechanisms. Cardiovasc Hematol Disord Drug Targets. (2012) 12:126–32. 10.2174/1871529x11202020126 23030445

[5] Kuter DJ. The biology of thrombopoietin and thrombopoietin receptor agonists. Int J Hematol. (2013) 98:10–23. 10.1007/s12185-013-1382-0 23821332

[6] Xu M Li J Neves MAD Zhu G Carrim N Yu R. GPIbalpha is required for platelet-mediated hepatic thrombopoietin generation. Blood. (2018) 132:622–34. 10.1182/blood-2017-12-820779 29794068

[7] Ng P Kauppi M Metcalf D Hyland CD Josefsson EC Lebois M. Mpl expression on megakaryocytes and platelets is dispensable for thrombopoiesis but essential to prevent myeloproliferation. Proc Natl Acad Sci USA. (2014) 111:5884–9. 10.1073/pnas.1404354111 24711413 PMC4000844

[8] Wright J The origin and nature of blood plates. Boston Med Surg J. (1906) 154:643–5. 10.1056/NEJM190606071542301

[9] Junt T Schulze H Chen Z Massberg S Goerge T Krueger A. Dynamic visualization of thrombopoiesis within bone marrow. Science. (2007) 317:1767–70. 10.1126/science.1146304 17885137

[10] Lefrancais E Ortiz-Munoz G Caudrillier A Mallavia B Liu F Sayah DM. The lung is a site of platelet biogenesis and a reservoir for haematopoietic progenitors. Nature. (2017) 544:105–9. 10.1038/nature21706 28329764 PMC5663284

[11] Chen ZY Oswald BE Sullivan JA Dahmani FZ Pasman Y Liu Z Platelet physiology and immunology: pathogenesis and treatment of classcial and non-classical fetal and neonatal alloimmune thrombocytopenia. Ann Blood. (2019) 4:29 10.21037/aob.2019.12.04

[12] Wang Y Gallant RC Ni H. Extracellular matrix proteins in the regulation of thrombus formation. Curr Opin Hematol. (2016) 23:280–7. 10.1097/MOH.0000000000000237 26871252

[13] Brass LF Diamond SL Stalker TJ. Platelets and hemostasis: a new perspective on an old subject. Blood Adv. (2016) 1:5–9. 10.1182/bloodadvances.2016000059 29296690 PMC5744048

[14] Ruggeri ZM. Mechanisms initiating platelet thrombus formation. Thromb Haemost. (1997) 78:611–6. 9198225

[15] Xu XR Carrim N Neves MA McKeown T Stratton TW Coelho RM. Platelets and platelet adhesion molecules: novel mechanisms of thrombosis and anti-thrombotic therapies. Thromb J. (2016) 14(Suppl. 1):29. 10.1186/s12959-016-0100-6 27766055 PMC5056500

[16] Kapur R Zufferey A Boilard E Semple JW. Nouvelle cuisine: platelets served with inflammation. J Immunol. (2015) 194:5579–87. 10.4049/jimmunol.1500259 26048965

[17] Kapur R Semple JW. Platelets as immune-sensing cells. Blood Adv. (2016) 1:10–14. 10.1182/bloodadvances.2016000067 29296691 PMC5744049

[18] Gaertner F Massberg S. Patrolling the vascular borders: platelets in immunity to infection and cancer. Nat Rev Immunol. (2019) 19:747–60. 10.1038/s41577-019-0202-z 31409920

[19] Yeaman MR. Platelets: at the nexus of antimicrobial defence. Nat Rev Microbiol. (2014) 12:426–37. 10.1038/nrmicro3269 24830471

[20] Xu XR Yousef GM Ni H. Cancer and platelet crosstalk: opportunities and challenges for aspirin and other antiplatelet agents. Blood. (2018) 131:1777–89. 10.1182/blood-2017-05-743187 29519806

[21] Vieira-de-Abreu A Campbell RA Weyrich AS Zimmerman GA. Platelets: versatile effector cells in hemostasis, inflammation, and the immune continuum. Semin Immunopathol. (2012) 34:5–30. 10.1007/s00281-011-0286-4 21818701 PMC4334392

[22] Murphy J Bijl N Yvan-Charvet L Welch CB Bhagwat N Reheman A. Cholesterol efflux in megakaryocyte progenitors suppresses platelet production and thrombocytosis. Nat Med. (2013) 19:586–94. 10.1038/nm.3150 23584088 PMC3683965

[23] Li C Li J Li Y Lang S Yougbare I Zhu G. Crosstalk between platelets and the immune system: old systems with new discoveries. Adv Hematol. (2012) 2012:384685. 10.1155/2012/384685 23008717 PMC3447344

[24] Assinger A. Platelets and infection - an emerging role of platelets in viral infection. Front Immunol. (2014) 5:649. 10.3389/fimmu.2014.00649 25566260 PMC4270245

[25] Guo L Rondina MT. The era of thromboinflammation: platelets are dynamic sensors and effector cells during infectious diseases. Front Immunol. (2019) 10:2204. 10.3389/fimmu.2019.02204 31572400 PMC6753373

[26] Franchini M Veneri D Lippi G. Thrombocytopenia and infections. Expert Rev Hematol. (2017) 10:99–106. 10.1080/17474086.2017.1271319 27936979

[27] Norgaard M Jensen AO Engebjerg MC Farkas DK Thomsen RW Cha S. Long-term clinical outcomes of patients with primary chronic immune thrombocytopenia: a danish population-based cohort study. Blood. (2011) 117:3514–20. 10.1182/blood-2010-10-312819 21263148

[28] McMorran J Marshall VM de Graaf C Drysdale KE Shabbar M Smyth GK. Platelets kill intraerythrocytic malarial parasites and mediate survival to infection. Science. (2009) 323:797–800. 10.1126/science.1166296 19197068

[29] Clark SR Ma AC Tavener SA McDonald B Goodarzi Z Kelly MM. Platelet TLR4 activates neutrophil extracellular traps to ensnare bacteria in septic blood. Nat Med. (2007) 13:463–9. 10.1038/nm1565 17384648

[30] Qu M Liu Q Zhao HG Peng J Ni H Hou M. Low platelet count as risk factor for infections in patients with primary immune thrombocytopenia: a retrospective evaluation. Ann Hematol. (2018) 97:1701–6. 10.1007/s00277-018-3367-9 29777278 PMC6097778

[31] van der Poll T van de Veerdonk FL Scicluna BP Netea MG. The immunopathology of sepsis and potential therapeutic targets. Nat Rev Immunol. (2017) 17:407–20. 10.1038/nri.2017.36 28436424

[32] Middleton EA Rowley JW Campbell RA Grissom CK Brown SM Beesley SJ. Sepsis alters the transcriptional and translational landscape of human and murine platelets. Blood. (2019) 134:911–23. 10.1182/blood.2019000067 31366617 PMC6753624

[33] Lei X Reheman A Hou Y Zhou H Wang Y Marshall AH. Anfibatide, a novel GPIb complex antagonist, inhibits platelet adhesion and thrombus formation *in vitro* and *in vivo* in murine models of thrombosis. Thromb Haemost. (2014) 111:279–89. 10.1160/TH13-06-0490 24172860

[34] Ni H Denis CV Subbarao S Degen JL Sato TN Hynes RO. Persistence of platelet thrombus formation in arterioles of mice lacking both von willebrand factor and fibrinogen. J Clin Invest. (2000) 106:385–92. 10.1172/JCI9896 10930441 PMC314330

[35] Strony J Beaudoin A Brands D Adelman B. Analysis of shear stress and hemodynamic factors in a model of coronary artery stenosis and thrombosis. Am J Physiol. (1993) 265(5 Pt 2):H1787–96. 10.1152/ajpheart.1993.265.5.H1787 8238592

[36] Ni H Freedman J. Platelets in hemostasis and thrombosis: role of integrins and their ligands. Transfus Apher Sci. (2003) 28:257–64. 10.1016/S1473-0502(03)00044-2 12725952

[37] Yang H Reheman A Chen P Zhu G Hynes RO Freedman J. Fibrinogen and von willebrand factor-independent platelet aggregation *in vitro* and *in vivo*. J Thromb Haemost. (2006) 4:2230–7. 10.1111/j.1538-7836.2006.02116.x 16824188

[38] Reheman A Yang H Zhu G Jin W He F Spring CM. Plasma fibronectin depletion enhances platelet aggregation and thrombus formation in mice lacking fibrinogen and von willebrand factor. Blood. (2009) 113:1809–17. 10.1182/blood-2008-04-148361 19036705

[39] Dunne E Spring CM Reheman A Jin W Berndt MC Newman DK. Cadherin 6 has a functional role in platelet aggregation and thrombus formation. Arterioscler Thromb Vasc Biol. (2012) 32:1724–31. 10.1161/ATVBAHA.112.250464 22539596 PMC4155514

[40] Wang Y Reheman A Spring CM Kalantari J Marshall AH Wolberg AS. Plasma fibronectin supports hemostasis and regulates thrombosis. J Clin Invest. (2014) 124:4281–93. 10.1172/JCI74630 25180602 PMC4191008

[41] Hou Y Carrim N Wang Y Gallant RC Marshall A Ni H. Platelets in hemostasis and thrombosis: novel mechanisms of fibrinogen-independent platelet aggregation and fibronectin-mediated protein wave of hemostasis. J Biomed Res. (2015) 29:437–44. 10.7555/JBR.29.20150121 26541706 PMC4662204

[42] Xu X Wu J Zhai Z Zhou R Wang X Wang H. A novel fibrinogen Bbeta chain frameshift mutation in a patient with severe congenital hypofibrinogenaemia. Thromb Haemost. (2006) 95:931–5. 10.1160/TH06-01-0020 16732370

[43] Zhai Z Wu J Xu X Ding K Ni R Hu W. Fibrinogen controls human platelet fibronectin internalization and cell-surface retention. J Thromb Haemost. (2007) 5:1740–6. 10.1111/j.1538-7836.2007.02625.x 17596138

[44] Roberts HR Hoffman M Monroe DM. A cell-based model of thrombin generation. Semin Thromb Hemost. (2006) 32(Suppl. 1):32–8. 10.1055/s-2006-939552 16673264

[45] Nurden T Caen JP. Specific roles for platelet surface glycoproteins in platelet function. Nature. (1975) 255:720–2. 10.1038/255720a0 1169691

[46] Ruggeri ZM. Platelets in atherothrombosis. Nat Med. (2002) 8:1227–34. 10.1038/nm1102-1227 12411949

[47] Koupenova M Clancy L Corkrey HA Freedman JE. Circulating platelets as mediators of immunity, inflammation, and thrombosis. Circ Res. (2018) 122:337–51. 10.1161/CIRCRESAHA.117.310795 29348254 PMC5777300

[48] Reheman A Xu X Reddy EC Ni H. Targeting activated platelets and fibrinolysis: hitting two birds with one stone. Circ Res. (2014) 114:1070–3. 10.1161/CIRCRESAHA.114.303600 24677231

[49] Li C Piran S Chen P Lang S Zarpellon A Jin JW. The maternal immune response to fetal platelet GPIbα causes frequent miscarriage in mice that can be prevented by intravenous IgG and anti-FcRn therapies. J Clin Invest. (2011) 121:4537–47. 10.1172/JCI57850 22019589 PMC3204841

[50] Hughes GR. Thrombosis, abortion, cerebral disease, and the lupus anticoagulant. Br Med J. (1983) 287:1088–9. 10.1136/bmj.287.6399.1088 6414579 PMC1549319

[51] Bates SM. Consultative hematology: the pregnant patient pregnancy loss. Hematol Am Soc Hematol Educ Program. (2010) 2010:166–72. 10.1182/asheducation-2010.1.166 21239788

[52] Bertozzi C Hess PR Kahn ML. Platelets: covert regulators of lymphatic development. Arterioscler Thromb Vasc Biol. (2010) 30:2368–71. 10.1161/ATVBAHA.110.217281 21071706 PMC2994722

[53] Herzog H Fu J Wilson SJ Hess PR Sen A McDaniel JM. Podoplanin maintains high endothelial venule integrity by interacting with platelet CLEC-2. Nature. (2013) 502:105–9. 10.1038/nature12501 23995678 PMC3791160

[54] Bertozzi C Schmaier AA Mericko P Hess PR Zou Z Chen M. Platelets regulate lymphatic vascular development through CLEC-2-SLP-76 signaling. Blood. (2010) 116:661–70. 10.1182/blood-2010-02-270876 20363774 PMC3324297

[55] Lowe KL Finney BA Deppermann C Hagerling R Gazit SL Frampton J. Podoplanin and CLEC-2 drive cerebrovascular patterning and integrity during development. Blood. (2015) 125:3769–77. 10.1182/blood-2014-09-603803 25908104 PMC4463737

[56] Osada M Inoue O Ding G Shirai T Ichise H Hirayama K. Platelet activation receptor CLEC-2 regulates blood/lymphatic vessel separation by inhibiting proliferation, migration, and tube formation of lymphatic endothelial cells. J Biol Chem. (2012) 287:22241–52. 10.1074/jbc.M111.329987 22556408 PMC3381185

[57] Zhang S Zhang S Hu L Zhai L Xue R Ye J. Nucleotide-binding oligomerization domain 2 receptor is expressed in platelets and enhances platelet activation and thrombosis. Circulation. (2015) 131:1160–70. 10.1161/CIRCULATIONAHA.114.013743 25825396 PMC4382913

[58] Aslam R Speck ER Kim M Crow AR Bang KW Nestel FP. Platelet Toll-like receptor expression modulates lipopolysaccharide-induced thrombocytopenia and tumor necrosis factor-alpha production *in vivo*. Blood. (2006) 107:637–41. 10.1182/blood-2005-06-2202 16179373

[59] Clemetson KJ Clemetson JM Proudfoot AE Power CA Baggiolini M Wells TN. Functional expression of CCR1, CCR3, CCR4, and CXCR4 chemokine receptors on human platelets. Blood. (2000) 96:4046–54. 11110672

[60] Linge P Fortin PR Lood C Bengtsson AA Boilard E. The non-haemostatic role of platelets in systemic lupus erythematosus. Nat Rev Rheumatol. (2018) 14:195–213. 10.1038/nrrheum.2018.38 29559714

[61] Kopp HG Placke T Salih HR. Platelet-derived transforming growth factor-beta down-regulates NKG2D thereby inhibiting natural killer cell antitumor reactivity. Cancer Res. (2009) 69:7775–83. 10.1158/0008-5472.CAN-09-2123 19738039

[62] Elzey BD Tian J Jensen RJ Swanson AK Lees JR Lentz SR. Platelet-mediated modulation of adaptive immunity. A communication link between innate and adaptive immune compartments. Immunity. (2003) 19:9–19. 10.1016/s1074-7613(03)00177-8 12871635

[63] Elzey BD Ratliff TL Sowa JM Crist SA. Platelet CD40L at the interface of adaptive immunity. Thromb Res. (2011) 127:180–3. 10.1016/j.thromres.2010.10.011 21075431 PMC3073541

[64] Yang H Lang S Zhai Z Li L Kahr WH Chen P. Fibrinogen is required for maintenance of platelet intracellular and cell-surface P-selectin expression. Blood. (2009) 114:425–36. 10.1182/blood-2008-03-145821 19332769

[65] Diacovo TG Puri KD Warnock RA Springer TA von Andrian HU. Platelet-mediated lymphocyte delivery to high endothelial venules. Science. (1996) 273:252–5. 10.1126/science.273.5272.252 8662511

[66] Rachidi S Metelli A Riesenberg B Wu BX Nelson MH Wallace C. Platelets subvert T cell immunity against cancer via GARP-TGFbeta axis. Sci Immunol. (2017) 2:eaai7911. 10.1126/sciimmunol.aai7911 28763790 PMC5539882

[67] Bao W Bussel JB Heck S He W Karpoff M Boulad N. Improved regulatory T-cell activity in patients with chronic immune thrombocytopenia treated with thrombopoietic agents. Blood. (2010) 116:4639–45. 10.1182/blood-2010-04-281717 20688957 PMC2996119

[68] Cerutti M Rescigno M. The biology of intestinal immunoglobulin A responses. Immunity. (2008) 28:740–50. 10.1016/j.immuni.2008.05.001 18549797 PMC3057455

[69] Li C Lam E Perez-Shibayama C Ward LA Zhang J Lee D. Early-life programming of mesenteric lymph node stromal cell identity by the lymphotoxin pathway regulates adult mucosal immunity. Sci Immunol. (2019) 4:eaax1027. 10.1126/sciimmunol.aax1027 31862865

[70] Chen W Jin W Hardegen N Lei KJ Li L Marinos N. Conversion of peripheral CD4+CD25- naive T cells to CD4+CD25+ regulatory T cells by TGF-beta induction of transcription factor Foxp3. J Exp Med. (2003) 198:1875–86. 10.1084/jem.20030152 14676299 PMC2194145

[71] Fantini MC Becker C Monteleone G Pallone F Galle PR Neurath MF. Cutting edge: TGF-beta induces a regulatory phenotype in CD4+CD25- T cells through Foxp3 induction and down-regulation of Smad7. J Immunol. (2004) 172:5149–53. 10.4049/jimmunol.172.9.5149 15100250

[72] Wan YY Flavell AR. 'Yin-Yang' functions of transforming growth factor-beta and T regulatory cells in immune regulation. Immunol Rev. (2007) 220:199–213. 10.1111/j.1600-065X.2007.00565.x 17979848 PMC2614905

[73] Kraemer BF Campbell RA Schwertz H Cody MJ Franks Z Tolley ND. Novel anti-bacterial activities of beta-defensin 1 in human platelets: suppression of pathogen growth and signaling of neutrophil extracellular trap formation. PLoS Pathog. (2011) 7:e1002355. 10.1371/journal.ppat.1002355 22102811 PMC3213094

[74] White JG. Platelets are covercytes, not phagocytes: uptake of bacteria involves channels of the open canalicular system. Platelets. (2005) 16:121–31. 10.1080/09537100400007390 15823869

[75] Gaertner G Ahmad Z Rosenberger G Fan S Nicolai L Busch B. Migrating platelets are mechano-scavengers that collect and bundle bacteria. Cell. (2017) 171:1368–82.e23. 10.1016/j.cell.2017.11.001 29195076

[76] Kho S Barber BE Johar E Andries B Poespoprodjo JR Kenangalem E. Platelets kill circulating parasites of all major plasmodium species in human malaria. Blood. (2018) 132:1332–44. 10.1182/blood-2018-05-849307 30026183 PMC6161646

[77] McDonald B Urrutia R Yipp BG Jenne CN Kubes P. Intravascular neutrophil extracellular traps capture bacteria from the bloodstream during sepsis. Cell Host Microbe. (2012) 12:324–33. 10.1016/j.chom.2012.06.011 22980329

[78] Love MS Millholland MG Mishra S Kulkarni S Freeman KB Pan W. Platelet factor 4 activity against falciparum P, and its translation to nonpeptidic mimics as antimalarials. Cell Host Microbe. (2012) 12:815–23. 10.1016/j.chom.2012.10.017 23245326 PMC3638032

[79] Campbell RA Schwertz H Hottz ED Rowley JW Manne BK Washington AV. Human megakaryocytes possess intrinsic antiviral immunity through regulated induction of IFITM3. Blood. (2019) 133:2013–26. 10.1182/blood-2018-09-873984 30723081 PMC6509546

[80] Simon Y Sutherland MR Pryzdial EL. Dengue virus binding and replication by platelets. Blood. (2015) 126:378–85. 10.1182/blood-2014-09-598029 25943787 PMC4826145

[81] Rondina MT Weyrich AS. Dengue virus pirates human platelets. Blood. (2015) 126:286–7. 10.1182/blood-2015-05-647362 26185116 PMC4504944

[82] Banerjee M Huang Y Joshi S Popa GJ Mendenhall MD Wang QJ. Platelets endocytose viral particles and are activated via TLR (toll-like receptor) signaling. Arterioscler Thromb Vasc Biol. (2020) 40:1635–50. 10.1161/ATVBAHA.120.314180 32434410 PMC7316618

[83] Flaujac C Boukour S Cramer-Borde E. Platelets and viruses: an ambivalent relationship. Cell Mol Life Sci. (2010) 67:545–56. 10.1007/s00018-009-0209-x 20012669 PMC11115580

[84] Henn V Slupsky JR Grafe M Anagnostopoulos I Forster R Muller-Berghaus G. CD40 ligand on activated platelets triggers an inflammatory reaction of endothelial cells. Nature. (1998) 391:591–4. 10.1038/35393 9468137

[85] Wagner D Frenette PS. The vessel wall and its interactions. Blood. (2008) 111:5271–81. 10.1182/blood-2008-01-078204 18502843 PMC2396724

[86] Mayadas TN Johnson RC Rayburn H Hynes RO Wagner DD. Leukocyte rolling and extravasation are severely compromised in P selectin-deficient mice. Cell. (1993) 74:541–54. 10.1016/0092-8674(93)80055-j 7688665

[87] Martins P van Gils JM Mol A Hordijk PL Zwaginga JJ. Platelet binding to monocytes increases the adhesive properties of monocytes by up-regulating the expression and functionality of beta1 and beta2 integrins. J Leukoc Biol. (2006) 79:499–507. 10.1189/jlb.0605318 16415171

[88] Koenen RR von Hundelshausen P Nesmelova IV Zernecke A Liehn EA Sarabi A. Disrupting functional interactions between platelet chemokines inhibits atherosclerosis in hyperlipidemic mice. Nat Med. (2009) 15:97–103. 10.1038/nm.1898 19122657

[89] Grommes J Alard JE Drechsler M Wantha S Morgelin M Kuebler WM. Disruption of platelet-derived chemokine heteromers prevents neutrophil extravasation in acute lung injury. Am J Respir Crit Care Med. (2012) 185:628–36. 10.1164/rccm.201108-1533OC 22246174 PMC3326286

[90] Wong CH Jenne CN Petri B Chrobok NL Kubes P. Nucleation of platelets with blood-borne pathogens on Kupffer cells precedes other innate immunity and contributes to bacterial clearance. Nat Immunol. (2013) 14:785–92. 10.1038/ni.2631 23770641 PMC4972575

[91] Sorvillo N Cherpokova D Martinod K Wagner DD. Extracellular DNA NET-works with dire consequences for health. Circ Res. (2019) 125:470–88. 10.1161/CIRCRESAHA.119.314581 31518165 PMC6746252

[92] Slaba I Wang J Kolaczkowska E McDonald B Lee WY Kubes P. Imaging the dynamic platelet-neutrophil response in sterile liver injury and repair in mice. Hepatology. (2015) 62:1593–605. 10.1002/hep.28003 26202541

[93] Belz T Shortman K Bevan MJ Heath WR. CD8alpha+ dendritic cells selectively present MHC class I-restricted noncytolytic viral and intracellular bacterial antigens *in vivo*. J Immunol. (2005) 175:196–200. 10.4049/jimmunol.175.1.196 15972648 PMC2778481

[94] Verschoor A Neuenhahn M Navarini AA Graef P Plaumann A Seidlmeier A. A platelet-mediated system for shuttling blood-borne bacteria to CD8alpha+ dendritic cells depends on glycoprotein GPIb and complement C3. Nat Immunol. (2011) 12:1194–201. 10.1038/ni.2140 22037602

[95] Gerdes N Zhu L Ersoy M Hermansson A Hjemdahl P Hu H. Platelets regulate CD4^+^ T-cell differentiation via multiple chemokines in humans. Thromb Haemost. (2011) 106:353–62. 10.1160/TH11-01-0020 21655676

[96] Liu CY Battaglia M Lee SH Sun QH Aster RH Visentin GP. Platelet factor 4 differentially modulates CD4+CD25+ (regulatory) versus CD4+CD25- (nonregulatory) T cells. J Immunol. (2005) 174:2680–6. 10.4049/jimmunol.174.5.2680 15728475

[97] Li C Irrazabal T So CC Berru M Du L Lam E. The H2B deubiquitinase Usp22 promotes antibody class switch recombination by facilitating non-homologous end joining. Nat Commun. (2018) 9:1006. 10.1038/s41467-018-03455-x 29520062 PMC5843634

[98] Fuchs TA Brill A Duerschmied D Schatzberg D Monestier M Myers DD. Extracellular DNA traps promote thrombosis. Proc Natl Acad Sci USA. (2010) 107:15880–5. 10.1073/pnas.1005743107 20798043 PMC2936604

[99] Swinkels M Rijkers M Voorberg J Vidarsson G Leebeek FWG Jansen AJG. Emerging concepts in immune thrombocytopenia. Front Immunol. (2018) 9:880. 10.3389/fimmu.2018.00880 29760702 PMC5937051

[100] Li C Chen P Vadasz B Ma L Zhou H Lang S. Co-stimulation with LPS or Poly I:C markedly enhances the anti-platelet immune response and severity of fetal and neonatal alloimmune thrombocytopenia. Thromb Haemost. (2013) 110:1250–8. 10.1160/TH13-04-0354 24067944

[101] Vadasz B Chen P Yougbare I Zdravic D Li J Li C. Platelets and platelet alloantigens: lessons from human patients and animal models of fetal and neonatal alloimmune thrombocytopenia. Genes Dis. (2015) 2:173–85. 10.1016/j.gendis.2015.02.003 28345015 PMC5362271

[102] Monroe DM Hoffman M Roberts HR Platelets and thrombin generation. Arterioscler Thromb Vasc Biol. (2002) 22:1381–9. 10.1161/01.atv.0000031340.68494.34 12231555

[103] Swieringa F Spronk HMH Heemskerk JWM van der Meijden PEJ. Integrating platelet and coagulation activation in fibrin clot formation. Res Pract Thromb Haemost. (2018) 2:450–60. 10.1002/rth2.12107 30046749 PMC6046596

[104] Andonegui G Kerfoot SM McNagny K Ebbert KV Patel KD Kubes P. Platelets express functional Toll-like receptor-4. Blood. (2005) 106:2417–23. 10.1182/blood-2005-03-0916 15961512

[105] Pang X Zhang R Cheng G. Progress towards understanding the pathogenesis of dengue hemorrhagic fever. Virol Sin. (2017) 32:16–22. 10.1007/s12250-016-3855-9 27853992 PMC6702245

[106] Hottz D Oliveira MF Nunes PC Nogueira RM Valls-de-Souza R Da Poian AT. Dengue induces platelet activation, mitochondrial dysfunction and cell death through mechanisms that involve DC-SIGN and caspases. J Thromb Haemost. (2013) 11:951–62. 10.1111/jth.12178 23433144 PMC3971842

[107] Nedeva C Menassa J Puthalakath H. Sepsis: inflammation is a necessary evil. Front Cell Dev Biol. (2019) 7:108. 10.3389/fcell.2019.00108 31281814 PMC6596337

[108] Davizon-Castillo P McMahon B Aguila S Bark D Ashworth K Allawzi A. TNF-alpha-driven inflammation and mitochondrial dysfunction define the platelet hyperreactivity of aging. Blood. (2019) 134:727–40. 10.1182/blood.2019000200 31311815 PMC6716075

[109] Boilard E Pare G Rousseau M Cloutier N Dubuc I Levesque T. Influenza virus H1N1 activates platelets through FcgammaRIIA signaling and thrombin generation. Blood. (2014) 123:2854–63. 10.1182/blood-2013-07-515536 24665136

[110] Powers ME Becker RE Sailer A Turner JR Bubeck Wardenburg J. Synergistic action of staphylococcus aureus alpha-toxin on platelets and myeloid lineage cells contributes to lethal sepsis. Cell Host Microbe. (2015) 17:775–87. 10.1016/j.chom.2015.05.011 26067604 PMC4642999

[111] de Haas CJC Weeterings C Vughs MM de Groot PG Van Strijp JA Lisman T. Staphylococcal superantigen-like 5 activates platelets and supports platelet adhesion under flow conditions, which involves glycoprotein Ibalpha and alpha IIb beta 3. J Thromb Haemost. (2009) 7:1867–74. 10.1111/j.1538-7836.2009.03564.x 19656281

[112] Waller K Sage T Kumar C Carr T Gibbins JM Clarke SR. Staphylococcus aureus lipoteichoic acid inhibits platelet activation and thrombus formation via the Paf receptor. J Infect Dis. (2013) 208:2046–57. 10.1093/infdis/jit398 23911710 PMC3836464

[113] Kraemer F Campbell RA Schwertz H Franks ZG de Abreu AV Grundler K. Bacteria differentially induce degradation of Bcl-xL, a survival protein, by human platelets. Blood. (2012) 120:5014–20. 10.1182/blood-2012-04-420661 23086749 PMC3525025

[114] Nagata S Suzuki J Segawa K Fujii T. Exposure of phosphatidylserine on the cell surface. Cell Death Differ. (2016) 23:952–61. 10.1038/cdd.2016.7 26891692 PMC4987739

[115] Gando S Kameue T Nanzaki S Nakanishi Y. Disseminated intravascular coagulation is a frequent complication of systemic inflammatory response syndrome. Thromb Haemost. (1996) 75:224–8. 8815564

[116] Levi M Ten Cate H. Disseminated intravascular coagulation. N Engl J Med. (1999) 341:586–92. 10.1056/NEJM199908193410807 10451465

[117] Wright F Blanchette VS Wang H Arya N Petric M Semple JW. Characterization of platelet-reactive antibodies in children with varicella-associated acute immune thrombocytopenic purpura (ITP). Br J Haematol. (1996) 95:145–52. 10.1046/j.1365-2141.1996.d01-1872.x 8857953

[118] Takahashi T Yujiri T Shinohara K Inoue Y Sato Y Fujii Y. Molecular mimicry by Helicobacter pylori CagA protein may be involved in the pathogenesis of H. pylori-associated chronic idiopathic thrombocytopenic purpura. Br J Haematol. (2004) 124:91–6. 10.1046/j.1365-2141.2003.04735.x 14675413

[119] Liebman H. Other immune thrombocytopenias. Semin Hematol. (2007) 44(4 Suppl. 5):S24–34. 10.1053/j.seminhematol.2007.11.004 18096469

[120] Li Z Nardi MA Karpatkin S. Role of molecular mimicry to HIV-1 peptides in HIV-1-related immunologic thrombocytopenia. Blood. (2005) 106:572–6. 10.1182/blood-2005-01-0243 15774614 PMC1895171

[121] Zhang W Nardi MA Borkowsky W Li Z Karpatkin S. Role of molecular mimicry of hepatitis C virus protein with platelet GPIIIa in hepatitis C-related immunologic thrombocytopenia. Blood. (2009) 113:4086–93. 10.1182/blood-2008-09-181073 19023115 PMC2673130

[122] Semple W Aslam R Kim M Speck ER Freedman J. Platelet-bound lipopolysaccharide enhances Fc receptor-mediated phagocytosis of IgG-opsonized platelets. Blood. (2007) 109:4803–5. 10.1182/blood-2006-12-062695 17299089

[123] Kapur R Heitink-Polle KM Porcelijn L Bentlage AE Bruin MC Visser R. C-reactive protein enhances IgG-mediated phagocyte responses and thrombocytopenia. Blood. (2015) 125:1793–802. 10.1182/blood-2014-05-579110 25548320

[124] Semple W. C-reactive protein boosts antibody-mediated platelet destruction. Blood. (2015) 125:1690–1. 10.1182/blood-2015-01-621219 25766565

[125] Severi E Hood DW Thomas GH. Sialic acid utilization by bacterial pathogens. Microbiology. (2007) 153(Pt 9):2817–22. 10.1099/mic.0.2007/009480-0 17768226

[126] Wasik BR Barnard KN Parrish CR. Effects of sialic acid modifications on virus binding and infection. Trends Microbiol. (2016) 24:991–1001. 10.1016/j.tim.2016.07.005 27491885 PMC5123965

[127] Syed S Hakala P Singh AK Lapatto HAK King SJ Meri S. Role of pneumococcal nana neuraminidase activity in peripheral blood. Front Cell Infect Microbiol. (2019) 9:218. 10.3389/fcimb.2019.00218 31297339 PMC6608562

[128] Riswari SF Tunjungputri RN Kullaya V Garishah FM Utari GSR Farhanah N. Desialylation of platelets induced by Von Willebrand Factor is a novel mechanism of platelet clearance in dengue. PLoS Pathog. (2019) 15:e1007500. 10.1371/journal.ppat.1007500 30849118 PMC6426266

[129] Li J van der Wal DE Zhu G Xu M Yougbare I Ma L. Desialylation is a mechanism of Fc-independent platelet clearance and a therapeutic target in immune thrombocytopenia. Nat Commun. (2015) 6:7737. 10.1038/ncomms8737 26185093 PMC4518313

[130] Grewal PK Uchiyama S Ditto D Varki N Le DT Nizet V. The ashwell receptor mitigates the lethal coagulopathy of sepsis. Nat Med. (2008) 14:648–55. 10.1038/nm1760 18488037 PMC2853759

[131] Kullaya V de Jonge MI Langereis JD van der Gaast-de Jongh CE Bull C Adema GJ. Desialylation of platelets by pneumococcal neuraminidase a induces ADP-dependent platelet hyperreactivity. Infect Immun. (2018) 86:e00213–18. 10.1128/IAI.00213-18 30037798 PMC6204724

[132] Keane C Tilley D Cunningham A Smolenski A Kadioglu A Cox D. Invasive *Streptococcus* pneumoniae trigger platelet activation via Toll-like receptor 2. J Thromb Haemost. (2010) 8:2757–65. 10.1111/j.1538-7836.2010.04093.x 20946179

[133] Shaim H McCaffrey P Trieu JA DeAnda A Yates SG. Evaluating the effects of oseltamivir phosphate on platelet counts: a retrospective review. Platelets. (2020) 31:1–5. 10.1080/09537104.2020.1714576 31931672

[134] Grewal PK Aziz PV Uchiyama S Rubio GR Lardone RD Le D. Inducing host protection in pneumococcal sepsis by preactivation of the Ashwell-Morell receptor. Proc Natl Acad Sci USA. (2013) 110:20218–23. 10.1073/pnas.1313905110 24284176 PMC3864324

[135] Deppermann C Kratofil RM Peiseler M David BA Zindel J Castanheira FVES. Macrophage galactose lectin is critical for kupffer cells to clear aged platelets. J Exp Med. (2020) 217:e20190723. 10.1084/jem.20190723 31978220 PMC7144524

[136] Stasi R Chia LW Kalkur P Lowe R Shannon MS. Pathobiology and treatment of hepatitis virus-related thrombocytopenia. Mediterr J Hematol Infect Dis. (2009) 1:e2009023. 10.4084/MJHID.2009.023 21415958 PMC3033122

[137] Clark B Noisakran S Onlamoon N Hsiao HM Roback J Villinger F. Multiploid CD61+ cells are the pre-dominant cell lineage infected during acute dengue virus infection in bone marrow. PLoS ONE. (2012) 7:e52902. 10.1371/journal.pone.0052902 23300812 PMC3531377

[138] Noisakran S Onlamoon N Hsiao HM Clark KB Villinger F Ansari AA. Infection of bone marrow cells by dengue virus *in vivo*. Exp Hematol. (2012) 40:250–9.e4. 10.1016/j.exphem.2011.11.011 22193689 PMC3415316

[139] Moses A Nelson J Bagby GC Jr. The influence of human immunodeficiency virus-1 on hematopoiesis. Blood. (1998) 91:1479–95. 9473211

[140] Gibellini D Clo A Morini S Miserocchi A Ponti C Re MC. Effects of human immunodeficiency virus on the erythrocyte and megakaryocyte lineages. World J Virol. (2013) 2:91–101. 10.5501/wjv.v2.i2.91 24175233 PMC3785048

[141] Haas S Hansson J Klimmeck D Loeffler D Velten L Uckelmann H. Inflammation-induced emergency megakaryopoiesis driven by hematopoietic stem cell-like megakaryocyte progenitors. Cell Stem Cell. (2015) 17:422–34. 10.1016/j.stem.2015.07.007 26299573

[142] Cines DB Cuker A Semple JW. Pathogenesis of immune thrombocytopenia. Presse Med. (2014) 43(4 Pt 2):e49–59. 10.1016/j.lpm.2014.01.010 24630266

[143] Provan D Stasi R Newland AC Blanchette VS Bolton-Maggs P Bussel JB. International consensus report on the investigation and management of primary immune thrombocytopenia. Blood. (2010) 115:168–86. 10.1182/blood-2009-06-225565 19846889

[144] Tao L Zeng Q Li J Xu M Wang J Pan Y. Platelet desialylation correlates with efficacy of first-line therapies for immune thrombocytopenia. J Hematol Oncol. (2017) 10:46. 10.1186/s13045-017-0413-3 28179000 PMC5304552

[145] Shao L Wu Y Zhou H Qin P Ni H Peng J Successful treatment with oseltamivir phosphate in a patient with chronic immune thrombocytopenia positive for anti-GPIb/IX autoantibody. Platelets. (2015) 26:495–7. 10.3109/09537104.2014.948838 25166956

[146] Zeng Q Zhu L Tao L Bao J Yang M Simpson EK. Relative efficacy of steroid therapy in immune thrombocytopenia mediated by anti-platelet GPIIbIIIa versus GPIbα antibodies. Am J Hematol. (2012) 87:206–8. 10.1002/ajh.22211 22139961

[147] Al-Samkari H Rosovsky RP Karp Leaf RS Smith DB Goodarzi K Fogerty AE. A modern reassessment of glycoprotein-specific direct platelet autoantibody testing in immune thrombocytopenia. Blood Adv. (2020) 4:9–18. 10.1182/bloodadvances.2019000868 31891657 PMC6960466

[148] Porcelijn L Huiskes E Oldert G Schipperus M Zwaginga JJ de Haas M. Detection of platelet autoantibodies to identify immune thrombocytopenia: state of the art. Br J Haematol. (2018) 182:423–6. 10.1111/bjh.15404 29808904

[149] Porcelijn L Schmidt DE van der Schoot CE Vidarsson G de Haas M Kapur R. Anti-glycoprotein Ibalpha autoantibodies do not impair circulating thrombopoietin levels in immune thrombocytopenia patients. Haematologica. (2020) 105:e172–4. 10.3324/haematol.2019.228908 31296573 PMC7109722

[150] Aslam R Kapur R Segel GB Guo L Zufferey A Ni H The spleen dictates platelet destruction, anti-platelet antibody production, and lymphocyte distribution patterns in a murine model of immune thrombocytopenia. Exp Hematol. (2016) 44:924–30.e1. 10.1016/j.exphem.2016.07.004 27417974

[151] Li Sullivan JA Ni H. Is platelet desialylation a novel biomarker and therapeutic target in immune thrombocytopenia? J Cell Immunol. (2020) 2:6–14.26185093

[152] Li Sullivan JA Ni H. Pathophysiology of immune thrombocytopenia. Curr Opin Hematol. (2018) 25:373–81. 10.1097/MOH.0000000000000447 30015642

[153] Olsson B Andersson PO Jernas M Jacobsson S Carlsson B Carlsson LM. T-cell-mediated cytotoxicity toward platelets in chronic idiopathic thrombocytopenic purpura. Nat Med. (2003) 9:1123–4. 10.1038/nm921 12937414

[154] Ma L Simpson E Li J Xuan M Xu M Baker L. CD8+ T cells are predominantly protective and required for effective steroid therapy in murine models of immune thrombocytopenia. Blood. (2015) 126:247–56. 10.1182/blood-2015-03-635417 26036802 PMC4505012

[155] McKenzie CG Guo L Freedman J Semple JW Cellular immune dysfunction in immune thrombocytopenia (ITP). Br J Haematol. (2013) 163:10–23. 10.1111/bjh.12480 23937260

[156] Zufferey A Kapur R Semple JW. Pathogenesis and therapeutic mechanisms in immune thrombocytopenia (ITP). J Clin Med. (2017) 6:16. 10.3390/jcm6020016 28208757 PMC5332920

[157] Chow L Aslam R Speck ER Kim M Cridland N Webster ML. A murine model of severe immune thrombocytopenia is induced by antibody- and CD8+ T cell-mediated responses that are differentially sensitive to therapy. Blood. (2010) 115:1247–53. 10.1182/blood-2009-09-244772 20007808

[158] Qiu J Liu X Li X Zhang X Han P Zhou H. CD8^+^ T cells induce platelet clearance in the liver via platelet desialylation in immune thrombocytopenia. Sci Rep. (2016) 6:27445. 10.1038/srep27445 27321376 PMC4913243

[159] Zhang F Chu X Wang L Zhu Y Li L Ma D. Cell-mediated lysis of autologous platelets in chronic idiopathic thrombocytopenic purpura. Eur J Haematol. (2006) 76:427–31. 10.1111/j.1600-0609.2005.00622.x 16480433

[160] Li S Wang L Zhao C Li L Peng J Hou M. CD8+ T cells suppress autologous megakaryocyte apoptosis in idiopathic thrombocytopenic purpura. Br J Haematol. (2007) 139:605–11. 10.1111/j.1365-2141.2007.06737.x 17979946

[161] Fahim M Monir E. Functional role of CD4+CD25+ regulatory T cells and transforming growth factor-beta1 in childhood immune thrombocytopenic purpura. Egypt J Immunol. (2006) 13:173–87. 17974160

[162] Liu B Zhao H Poon MC Han Z Gu D Xu M. Abnormality of CD4^+^CD25^+^ regulatory T cells in idiopathic thrombocytopenic purpura. Eur J Haematol. (2007) 78:139–43. 10.1111/j.1600-0609.2006.00780.x 17328716

[163] Ling Y Cao X Yu Z Ruan C. Circulating dendritic cells subsets and CD4+Foxp3+ regulatory T cells in adult patients with chronic ITP before and after treatment with high-dose dexamethasome. Eur J Haematol. (2007) 79:310–6. 10.1111/j.1600-0609.2007.00917.x 17692100

[164] Sakakura M Wada H Tawara I Nobori T Sugiyama T Sagawa N. Reduced Cd4+Cd25+ T cells in patients with idiopathic thrombocytopenic purpura. Thromb Res. (2007) 120:187–93. 10.1016/j.thromres.2006.09.008 17067661

[165] Yu J Heck S Patel V Levan J Yu Y Bussel JB. Defective circulating CD25 regulatory T cells in patients with chronic immune thrombocytopenic purpura. Blood. (2008) 112:1325–8. 10.1182/blood-2008-01-135335 18420827 PMC2515134

[166] Stasi R Cooper N Del Poeta G Stipa E Laura Evangelista M Abruzzese E. Analysis of regulatory T-cell changes in patients with idiopathic thrombocytopenic purpura receiving B cell-depleting therapy with rituximab. Blood. (2008) 112:1147–50. 10.1182/blood-2007-12-129262 18375792

[167] Olsson B Ridell B Carlsson L Jacobsson S Wadenvik H. Recruitment of T cells into bone marrow of ITP patients possibly due to elevated expression of VLA-4 and CX3CR1. Blood. (2008) 112:1078–84. 10.1182/blood-2008-02-139402 18519809

[168] Semple JW. ITP three R's: regulation, routing, rituximab. Blood. (2008) 112:927–8. 10.1182/blood-2008-05-155770 18684874

[169] Nishimoto T Kuwana M. CD4+CD25+Foxp3+ regulatory T cells in the pathophysiology of immune thrombocytopenia. Semin Hematol. (2013) 50(Suppl. 1):S43–9. 10.1053/j.seminhematol.2013.03.018 23664516

[170] Andersson O Stockelberg D Jacobsson S Wadenvik H. A transforming growth factor-beta1-mediated bystander immune suppression could be associated with remission of chronic idiopathic thrombocytopenic purpura. Ann Hematol. (2000) 79:507–13. 10.1007/s002770000177 11043422

[171] Andersson O Olsson A Wadenvik H. Reduced transforming growth factor-beta1 production by mononuclear cells from patients with active chronic idiopathic thrombocytopenic purpura. Br J Haematol. (2002) 116:862–7. 10.1046/j.0007-1048.2002.03345.x 11886393

[172] Aslam R Hu Y Gebremeskel S Segel GB Speck ER Guo L. Thymic retention of CD4+CD25+FoxP3+ T regulatory cells is associated with their peripheral deficiency and thrombocytopenia in a murine model of immune thrombocytopenia. Blood. (2012) 120:2127–32. 10.1182/blood-2012-02-413526 22760780

[173] Guo L Kapur R Aslam R Speck ER Zufferey A Zhao Y. CD20+ B-cell depletion therapy suppresses murine CD8+ T-cell-mediated immune thrombocytopenia. Blood. (2016) 127:735–8. 10.1182/blood-2015-06-655126 26556550

[174] Cines DB Bussel JB Liebman HA Luning Prak ET. The ITP syndrome: pathogenic and clinical diversity. Blood. (2009) 113:6511–21. 10.1182/blood-2009-01-129155 19395674 PMC2710913

[175] Johnsen J. Pathogenesis in immune thrombocytopenia: new insights. Hematol Am Soc Hematol Educ Program. (2012) 2012:306–12. 10.1182/asheducation-2012.1.306 23233597

[176] Ekstrand C Linder M Cherif H Kieler H Bahmanyar S. Increased susceptibility to infections before the diagnosis of immune thrombocytopenia. J Thromb Haemost. (2016) 14:807–14. 10.1111/jth.13267 26792007

[177] Goerge T Ho-Tin-Noe B Carbo C Benarafa C Remold-O'Donnell E Zhao BQ. Inflammation induces hemorrhage in thrombocytopenia. Blood. (2008) 111:4958–64. 10.1182/blood-2007-11-123620 18256319 PMC2384127

[178] Lu J Marjon KD Mold C Du Clos TW Sun PD. Pentraxins and Fc receptors. Immunol Rev. (2012) 250:230–8. 10.1111/j.1600-065X.2012.01162.x 23046133 PMC3471383

[179] Arepally GM. Heparin-induced thrombocytopenia. Blood. (2017) 129:2864–72. 10.1182/blood-2016-11-709873 28416511 PMC5445568

[180] Warkentin TE. HIT: still stringing us along. Blood. (2020) 135:1193–4. 10.1182/blood.2020005157 32271906

[181] Krauel K Potschke C Weber C Kessler W Furll B Ittermann T. Platelet factor 4 binds to bacteria, [corrected] inducing antibodies cross-reacting with the major antigen in heparin-induced thrombocytopenia. Blood. (2011) 117:1370–8. 10.1182/blood-2010-08-301424 20959601

[182] Krauel K Weber C Brandt S Zahringer U Mamat U Greinacher A. Platelet factor 4 binding to lipid A of Gram-negative bacteria exposes PF4/heparin-like epitopes. Blood. (2012) 120:3345–52. 10.1182/blood-2012-06-434985 22942185

[183] Bussel JB Primiani A. Fetal and neonatal alloimmune thrombocytopenia: progress and ongoing debates. Blood Rev. (2008) 22:33–52. 10.1016/j.blre.2007.09.002 17981381

[184] de Vos TW Winkelhorst D de Haas M Lopriore E Oepkes D. Epidemiology and management of fetal and neonatal alloimmune thrombocytopenia. Transfus Apher Sci. (2020) 59:102704. 10.1016/j.transci.2019.102704 31974030

[185] Zdravic D Yougbare I Vadasz B Li C Marshall AH Chen P. Fetal and neonatal alloimmune thrombocytopenia. Semin Fetal Neonatal Med. (2016) 21:19–27. 10.1016/j.siny.2015.12.004 26810319

[186] Roopenian DC Akilesh S. FcRn: the neonatal Fc receptor comes of age. Nat Rev Immunol. (2007) 7:715–25. 10.1038/nri2155 17703228

[187] Ni H Chen P Spring CM Sayeh E Semple JW Lazarus AH. A novel murine model of fetal and neonatal alloimmune thrombocytopenia: response to intravenous IgG therapy. Blood. (2006) 107:2976–83. 10.1182/blood-2005-06-2562 16317099 PMC1895387

[188] Chen P Li C Lang S Zhu G Reheman A Spring CM. Animal model of fetal and neonatal immune thrombocytopenia: role of neonatal Fc receptor in the pathogenesis and therapy. Blood. (2010) 116:3660–8. 10.1182/blood-2010-05-284919 20647570

[189] Tiller H Killie MK Husebekk A Skogen B Ni H Kjeldsen-Kragh J. Platelet antibodies and fetal growth: maternal antibodies against fetal platelet antigen 1a are strongly associated with reduced birthweight in boys. Acta Obstet Gynecol Scand. (2012) 91:79–86. 10.1111/j.1600-0412.2011.01269.x 21895612

[190] Bussel JB Zabusky MR Berkowitz RL McFarland JG. Fetal alloimmune thrombocytopenia. N Engl J Med. (1997) 337:22–6. 10.1056/NEJM199707033370104 9203427

[191] Bizzaro N Dianese G. Neonatal alloimmune amegakaryocytosis. Case report. Vox Sang. (1988) 54:112–4. 10.1111/j.1423-0410.1988.tb01627.x 3376462

[192] Kroll H Muntean W Kiefel V Giptner A Schluter C Santoso S. Anti Ko(a) as a cause of neonatal alloimmune thrombocytopenia. Beitr Infusionsther Transfusionsmed. (1994) 32:244–6. 9480100

[193] Al-Sheikh IH Khalifa M Rahi A Qadri MI Al Abad K. A rare case of neonatal alloimmune thrombocytopenia due to ANTI-HPA-2b. Ann Saudi Med. (1998) 18:547–9. 10.5144/0256-4947.1998.547 17344746

[194] Goldman M Trudel E Richard L Khalife S Spurll GM. Neonatal alloimmune thrombocytopenia due to anti-HPA-2b (anti-Koa). Immunohematology. (2003) 19:43–6. 15373693

[195] Davoren A Curtis BR Aster RH McFarland JG. Human platelet antigen-specific alloantibodies implicated in 1162 cases of neonatal alloimmune thrombocytopenia. Transfusion. (2004) 44:1220–5. 10.1111/j.1537-2995.2004.04026.x 15265127

[196] Yougbare I Lang S Yang H Chen P Zhao X Tai WS. Maternal anti-platelet beta3 integrins impair angiogenesis and cause intracranial hemorrhage. J Clin Invest. (2015) 125:1545–56. 10.1172/JCI77820 25774504 PMC4396473

[197] Yougbare I Tai WS Zdravic D Oswald BE Lang S Zhu G. Activated NK cells cause placental dysfunction and miscarriages in fetal alloimmune thrombocytopenia. Nat Commun. (2017) 8:224. 10.1038/s41467-017-00269-1 28794456 PMC5550461

[198] Huang PC Wang Y Li X Ren L Zhao J Hu Y. Clinical features of patients infected with 2019 novel coronavirus in Wuhan, China. Lancet. (2020) 395:497–506. 10.1016/S0140-6736(20)30183-5 31986264 PMC7159299

[199] Xu Z Shi L Wang Y Zhang J Huang L Zhang C. Pathological findings of COVID-19 associated with acute respiratory distress syndrome. Lancet Respir Med. (2020) 8:420–22. 10.1016/S2213-2600(20)30076-X 32085846 PMC7164771

[200] Guan WJ Ni ZY Hu Y Liang WH Ou CQ He JX. China medical treatment expert group for: clinical characteristics of coronavirus disease 2019 in China. N Engl J Med. (2020) 382:1708–20. 10.1056/NEJMoa2002032 32109013 PMC7092819

[201] Jiang Q Huang QF Xie WM Lv C Quan XQ. The association between severe COVID-19 and low platelet count: evidence from 31 observational studies involving 7613 participants. Br J Haematol. (2020) 190:e29–e33. 10.1111/bjh.16817 32420607

[202] Lippi G Plebani M Henry BM. Thrombocytopenia is associated with severe coronavirus disease 2019 (COVID-19) infections: a meta-analysis. Clin Chim Acta. (2020) 506:145–8. 10.1016/j.cca.2020.03.022 32178975 PMC7102663

[203] Bowles L Platton S Yartey N Dave M Lee K Hart DP. Lupus anticoagulant and abnormal coagulation tests in patients with covid-19. N Engl J Med. (2020) 383:288–90. 10.1056/NEJMc2013656 32369280 PMC7217555

[204] Bikdeli B Madhavan MV Jimenez D Chuich T Dreyfus I Driggin E. COVID-19 and thrombotic or thromboembolic disease: implications for prevention, antithrombotic therapy, and follow-up: JACC state-of-the-art review. J Am Coll Cardiol. (2020) 75:2950–73. 10.1016/j.jacc.2020.04.031 32311448 PMC7164881

[205] Klok FA Kruip MJHA van der Meer NJM Arbous MS Gommers D Kant KM Incidence of thrombotic complications in critically ill ICU patients with COVID-19. Thromb Res. (2020) 191:145–7. 10.1016/j.thromres.2020.04.013 32291094 PMC7146714

[206] Cui S Chen S Li X Liu S Wang F. Prevalence of venous thromboembolism in patients with severe novel coronavirus pneumonia. J Thromb Haemost. (2020) 18:1421–4. 10.1111/jth.14830 32271988 PMC7262324

[207] Luo W Yu H Gou Z Li X Sun Y Li J. Clinical pathology of critical patient with novel coronavirus pneumonia (COVID-19). [Preprint] (2020).32198776

